# Dream Land, Still They Come

**Published:** 1887-03

**Authors:** E. W. H. Beck


### DREAM LAND.—STILL THEY COME.

BY DR. E. W. H. BECK.

The following account of a remarkable dream was originally published in the January 8th issue of the *Religio-Philosophical Journal*, of Chicago, and was republished entire in Sunday's *New York World*, of January 16th. Its facts are closely related to a number of similar occurrences which we have laid before our readers the past twelve months:

DELPHI, IND.

On a bright November Sunday, some years since, Mr. and Mrs. Hiram Lovell, then residents of this place, but now of Burlington, this (Carroll) County, started in their buggy for a neighboring village, having with them their youngest child, then about twenty months old. The baby, prattling in its mother's lap, apparently well and strong, and enjoying the ride, suddenly startled its parents by struggling in a convulsion. This unlooked for event turned them homeward. Arriving there about sundown, another spasm occurred, and I, their family physician, was sent for. Relief was had for the night, but two more occurred on the following Monday morning and evening, each coming like a clap of thunder in a clear sky, as the child did not seem sick, but ate, laughed and played between the attacks as though nothing had occurred. Each day witnessed one or two convulsions. Of course, there was a cause, and in my search for and failure to find it, I was considerably nonplussed and perplexed. I had set aside by the method known to physicians as exclusion,—worms, teething, injesta, etc., as the cause, and settled down in the conviction that it was reflex irritation upon nerve centers, but what and where the impinging offense?

Friday morning I awoke from my sleep after having dreamed about my patient, and forthwith I related my dream to my wife. I now give it in full to the readers of the *Religio-Philosophical Journal*. It is the nut I want them to crack: I dreamed that I paid my usual morning visit to the sick child; that I passed through the hall, opened the door into the sitting-room, and saw the family seated at breakfast. Mrs. Murphy (now of Judsonia, Ark.), a lady boarder in the family, sat at the end of the table on my right; Mrs. Lovell sat on her right; Mr. L. next to her, and a gentleman boarder next to him; a stove on my left, and between the end of the table and the partition wall, was the cradle in which lay my little patient asleep—feet toward me. My dream took in this picture.

I then approached the cradle, deliberately lifted the left foot of the babe, saw on the sole (plantar surface), midway between toe and heel, a small red spot elevated two or three lines above the surface, and in circumference the size of a grain of corn.

I then deliberately and unhesitatingly thrust my thumb and finger into my right vest pocket, and taking out my thumb lancet, I punctured the rose-colored spot, when out came a needle three-fourths of an inch long, the eye first, when I said to Mrs. L., "Your child will have no more spasms." This was the dream, and now what followed was precisely as related in it, without the slightest exaggeration or shade of difference in fact.

Arising in the morning as usual, I ate my breakfast, and went immediately to the house of my patient, with the dream fresh in my mind, and with never a thought of its fulfillment. I had never been a dreamer, and am not a clairvoyant. I passed through the hall and opened the second door, to see the family at the table in the exact relative position mentioned above—baby, cradle and all! The only variation from the dream was that I sat down by the stove to warm my hands just inside the door to my left, and asking how the baby passed the night, was told by Mrs. L. that it had a good night—no spasms, and was now sleeping. I then narrated my dream in detail, causing a ripple of mirth and a hearty laugh from Mrs. L., who started up and toward the cradle, exclaiming, "We'll see now about this dream!" Lifting the foot, there surely was the red spot. I approached the cradle now, took the foot in my hand, and with my thumb lancet did puncture the spot, and a needle three-fourths of an inch long—eye first—did push out from its suppurating sack, the point broken off presumably before its entrance in the foot, and I did use the expression above stated to Mrs. L., and there was no further trouble.

Now, let us examine a few points relatively in the dream before we jump to the conclusion that it was the legitimate following of my anxiety for my little patient, or that the case was on my mind, sleeping or waking, and the result shadowed in tracings from conclusion to cause, from distil to proximate ends of the line.

I had never, up to this time, been in the house when the family were at their meal. I knew that there was a large kitchen in the rear of this sitting-room, and I supposéd they dined there. I saw in my dream very distinctly, and noted everything, even to the baking of buckwheat cakes on the parlor cook stove in this sitting room. I did not know that Mrs. M. boarded in the family, nor that they had a gentleman boarder. He was a stranger to me, never having seen him before that I remembered of, till in my dream, yet his face was familiar when I saw him at the table in the morning.

Another point: I invariably carried my thumb lancet in a small silver case in my left trousers pocket, always careful to cleanse and replace it after using. My dream made me take it out of my right vest pocket, without a thought of reaching for it in its usual place; possibly the only time I ever had it misplaced, and occasioned this time by the fact that the day before I abstracted blood from the arm of a stout lady in an epileptic seizure, and had to help hold her in her struggles, and hurriedly and thoughtlessly, the moment after letting blood, slipped it into my right vest pocket and forgot it. The vest pocket, and the lancet loose in it, was the last place I should have looked for it, in my waking state, for the case was still where I always carried it.

There is what is termed contiguous, continuous and remote sympathy in many of the diseases afflicting humanity. Is there such a law underlying the manifestations of mind force, consonant to, and in harmony with, our dual nature?—the shadow of substance, the fiction of fact! I have no theory in explanation more reasonable than that my spirit friends impressed my mind, during sleep, to see what they saw, in answer to my mental prayer, "Light, more Light."

